# Supplementary material for: Prevalence of intestinal parasite contamination in raw vegetables and school soil samples in Koh Yao, Phang Nga Province, southern Thailand
Source: PLoS One. 2025 Apr 1;20(4):e0320929. doi: 10.1371/journal.pone.0320929 (PMC11960992; doi:10.1371/journal.pone.0320929)
Supplement: Attachment — Submitted filename: Report on Reject Decision.pdf [file pone.0320929.s005.pdf]

Despite the authors' efforts to revise the manuscript, numerous issues remain unresolved:

1. **Abstract**: The mention of data being analyzed using IBM SPSS (version 26) is unnecessary in the abstract and should be moved to the “Statistical Analysis” section.
2. **Citations**: References need to be presented separately. For example:
   - "Maternal anemia in Bangladesh is associated with increased pregnancy complications (Ref#1-2), adverse birth outcomes (Ref#1-2), and higher risks of preterm birth (Ref#1-2), neonatal mortality (1-2 Ref), and perinatal mortality (1-2 Ref)"—these citations should be listed individually.
   - Similarly, in other sections:
     - Causes of anemia: "Anemia has multifactorial causes, including nutritional deficiencies (iron, folic acid, vitamin B12) (1-2 Ref), genetic conditions (thalassemia, sickle cell disease) (1-2 Ref), chronic diseases (cancer) (1-2 Ref), infections (malaria) (1-2 Ref), environmental pollutants (lead) (1-2 Ref), autoimmune disorders (1-2 Ref), malabsorption (1-2 Ref), hormonal changes (1-2 Ref), malnutrition (1-2 Ref), and socioeconomic factors like low maternal education and income (1-2 Ref), with menstruation in girls (1-2 Ref) often contributing to iron deficiency anemia."
     - Factors for anemia and undernutrition in adolescent girls: "Inadequate water (1-2 Ref), sanitation, and hygiene (WASH) practices (1-2 Ref), absence of deworming interventions (1-2 Ref), and limited access to television-based awareness programs (1-2 Ref)"—all require separate references.
3. **Validation and Citations**: The manuscript lacks citations on the validity of the food frequency questionnaire (FFQ), the formula used for BMI calculations, altitude adjustments for hemoglobin, and the cut-off values used.
4. **Statistical Analysis**: Unnecessary details are provided in the statistical analysis section, while critical information—such as the handling of skewed data and the application of appropriate statistical tests—is missing.
5. **Minor Errors**: Errors such as the use of “Tk” without spelling it out in full on first mention reflect a lack of attention to detail.
6. **Table 1**: The table is not properly aligned, lacks consistency in the number of digits after the decimal, and is not formatted correctly.
7. **Discussion**:
   - The first sentence of the discussion uses “JUST,” which seems informal and out of place.
   - Paragraph 1 of the discussion spans approximately two pages, which is excessively long.
   - The discussion lacks structure and does not reflect the revisions requested.
8. **Overall Quality**: The manuscript still lacks coherence and fails to meet the standards expected after two rounds of revisions.

Given these unresolved issues, the revision does not meet the necessary criteria, and manuscript does not meet our criteria for publication and must therefore be rejected.
