# Supplementary material for: Prevalence of intestinal parasite contamination in raw vegetables and school soil samples in Koh Yao, Phang Nga Province, southern Thailand
Source: PLoS One. 2025 Apr 1;20(4):e0320929. doi: 10.1371/journal.pone.0320929 (PMC11960992; doi:10.1371/journal.pone.0320929)
Supplement: Attachment — Submitted filename: Comments-PONE-D-24-44096.docx [file pone.0320929.s003.docx]

Dear authors,

With regard to the manuscript ID “PONE-D-24-44096”, I would like to express my general impression, comments and suggestion which will help enhance the impact of your current paper.

- **General impression of the research**

The study you have carried out reflects a significant contribution to the field of public health and parasitology. The methodological details, clear presentation of results, and insightful discussion provide a comprehensive correlation between the contamination of raw vegetables and soil in the public areas. Moreover, the analysis of soil sample in two different seasons also provides the general outlook regarding the chances of contamination of the edibles like the fresh vegetables, water and other food items.

- **Some point-by-point comments**

1. How about the trend of raising pigs in the study area, either intensively or semi-intensively or open system? As authors have mentioned the presence of *Ascaris lumbricoides* eggs in the examined vegetable samples. The *Ascaris lumbricoides* and *Ascaris suum* eggs (a soil transmitted helminth found in the pigs) are morphologically similar. Please describe clearly whether the source of *Ascaris* eggs was only from the human excreta?
2. Mention the trend of using of human excreta as the manure, which might be another supporting information for the source of contamination of vegetables.
3. **Line no: 76 and 229** – authors have mentioned earthworm. It must be ‘round worm’. Please make sure the exact term.
4. **Line no: 227**- authors have mentioned hookworm eggs, but the image of hookworm egg is not provided. Please provide it.
5. **Line no: 233** – authors have mentioned hookworm larva. Please describe in detail how did you confirm hookworm larva? Did you perform egg culture experiments? This might be the larva of other ‘strongyle nematodes’.
6. The image of *Toxocara* provided in the manuscript is not clear. Please provide with the clear image of it with the scale bar (if you have measured the dimension of eggs). Basically, in morphometric diagnosis of parasite eggs or larva, the scale bar is a must.
7. **Control measures:** Please provide strong recommendations for control measures of the soil borne parasites as described in the paper.
8. **Study limitations:** Provide a more in-depth discussion of potential limitations in your study. One of the limitations is lack of the simultaneous analysis of intestinal parasite infection trend among the school children or other public in the study area.
9. **Future Research Directions:** Suggest specific areas for future research.

……………………………….
