# Supplementary material for: Prevalence of intestinal parasite contamination in raw vegetables and school soil samples in Koh Yao, Phang Nga Province, southern Thailand
Source: PLoS One. 2025 Apr 1;20(4):e0320929. doi: 10.1371/journal.pone.0320929 (PMC11960992; doi:10.1371/journal.pone.0320929)
Supplement: Attachment — Submitted filename: Reviewers Report_version_2_PONE-D-24-44096.docx [file pone.0320929.s006.docx]

**Reviewer's Report	Date:** 2/8/2025

**Reviewer no. 1                                        	Version: 2**      
**Manuscript ID:** PONE-D-24-44096R1
**Title:** Prevalence of Intestinal Parasite Contamination in Raw Vegetables and School Soil Samples in Koh Yao, Phang Nga Province, Southern Thailand

**General Comments:**

The revised manuscript has incorporated most of the suggested improvements, particularly in enhancing the literature synthesis, restructuring the discussion, and improving the clarity of statistical reporting. The authors have strengthened the literature review, improved methodological clarity, and refined the discussion for better logical flow. The study provides valuable new data on the prevalence of intestinal parasites in raw vegetables and school soil, which has important public health implications. The methodology is sound, the results are clearly presented, and the discussion effectively interprets the findings in a relevant epidemiological context. Given these improvements, I **recommend the manuscript for acceptance.**

**Abstract:**

1. The revised abstract is well-structured and now includes associated factors along with prevalence results, addressing previous concerns.
2. The statistical significance of contamination differences is reported correctly.
3. The conclusion appropriately emphasizes the importance of hygiene practices and preventive measures.

**Introduction:**

1. The introduction now presents a strong and well-referenced literature review that establishes the knowledge gap and study rationale effectively.
2. The epidemiological context of soil-transmitted helminths and zoonotic parasites is well-explained, and the justification for selecting Koh Yao as the study site is clear.
3. The objectives are concise and align well with the study’s scope.

**Methods:**

1. The methodology is now well-detailed, ensuring reproducibility and transparency.
2. The authors have effectively clarified the sampling strategy, data collection methods, and statistical analyses used.
3. Hygiene assessment and ethical considerations are appropriately described.
4. The revisions ensure that the approach is robust and scientifically sound.

**Results:**

1. The results section is now well-organized and presents key findings with clarity.
2. The authors have successfully streamlined text to reduce redundancy while ensuring that all relevant data are effectively reported.
3. The statistical significance of contamination differences is well-presented, and tables are neatly formatted.

**Discussion:**

1. The discussion is more structured, with clearer explanations of contamination patterns.
2. The study’s findings are contextualized within public health frameworks, and the conclusions are directly supported by the data.
3. The contradiction regarding Chinese morning glory’s smooth surface and its contamination rate has been corrected.
4. The role of climate and environmental conditions in contamination variations is now discussed.

**Conclusion:**

1. The conclusion is now directly based on study findings, avoiding broad generalizations.
2. The emphasis on preventive strategies and the need for hygiene education is appropriate and actionable.
3. The recommendations for future research are well-stated and relevant.

**Comments for authors**
I commend the authors on their responsibility for reviewers' comments and overall, they adequately addressed my concerns.

Hence, I recommend acceptance of the manuscript.
